# Exploring social complexities of the COVID-19 pandemic on maternal anxiety: A mixed-methods observational cohort study

**DOI:** 10.18332/ejm/152200

**Published:** 2022-10-11

**Authors:** Michelle Anderson, Eleanor Pyart, Audrey Epstein, Rezan Abdul-Kadir

**Affiliations:** 1 Department of Obstetrics and Gynaecology, Royal Free Hospital, London, United Kingdom; 2 Department of Obstetrics and Gynaecology, Barnet Hospital, Chipping Barnet, United Kingdom

**Keywords:** pregnancy, anxiety, COVID-19, preterm birth

## Abstract

**INTRODUCTION:**

The aim of this mixed-methods, small-scale observational cohort study was to examine if anxiety in pregnant women increased during the COVID-19 pandemic and to examine the subsequent impact on birth outcomes and psychological well-being. This research was conducted across two hospital sites in North London, with participation from 194 pregnant women.

**METHODS:**

The GAD-7 questionnaire assessed for mild, moderate and high anxiety at one time point during the antenatal period and was repeated 6 weeks postnatally. Women with moderate to high scores on the GAD-7 were invited to participate in semi-structured interviews. The primary outcome measure was assessment of antenatal and postnatal anxiety. Secondary outcome measures assessed if women with moderate/high GAD-7 scores were more likely to develop a mental health condition during pregnancy, or up to 6 weeks postnatally, and if risk of preterm birth (<37 weeks gestation) and instrumental birth or cesarean section increased.

**RESULTS:**

Pearson’s correlation indicated a positive and significant correlation between the COVID-19 pandemic, and increased self-reported antenatal GAD-7 anxiety scores (r=0.47, n=194, p<0.001). GAD-7 scores were higher during pregnancy compared to the postnatal period [t(193)=4.63; p=0.001; 95% CI: 0.87–2.16]. Logistic regression did not show an increased likelihood of preterm birth [χ²(1, n=184)=0.999; p=0.971] or instrumental/cesarean section birth in women who scored moderately to highly on the antenatal GAD-7 [χ²(1, n=184)=2.73; p=0.165]. Qualitative analysis was carried out within a social constructionist framework and identified the following themes: anxiety, maternity care, social impact, and coping.

**CONCLUSIONS:**

Pregnant women self-reported an increase in antenatal anxiety during July 2020 to April 2021 of the COVID-19 pandemic. Moderate to high anxiety scores were not found to increase the likelihood of preterm birth and birth intervention or developing a mental health condition up to 6 weeks postnatally.

## INTRODUCTION

It is estimated that anxiety and depression affect approximately 10–15 per 100 pregnant women^1^. Maternal anxiety may develop simply because childbearing is a significant life transitioning event, and therefore could be considered a normal psychological component of the pregnancy continuum^2^. However, persistent anxiety is more concerning and associated with adverse effects for maternal mental health and birth outcome^3^, such as increased risk of preterm birth, low birth weight, birth intervention, and postnatal depression^2,4-9^. The consequences of maternal anxiety extend far beyond pregnancy and birth, with adverse psychosocial outcomes in the offspring and increased risk of emotional and behavioral problems in children^10-12^.

Since January 2020, pregnant women have navigated through the social complexities of the COVID-19 pandemic. Concerns about the morbidity and mortality associated with COVID-19 in pregnancy may have raised anxiety levels in women, especially during the early stages of the pandemic and could have been exacerbated by stringent lockdown measures, social distancing, and, consequently, disruption to maternity service provision. Lockdown measures are themselves key risk factors for deteriorating mental health^13^, mainly because of social isolation, financial stress, and increased instances of domestic abuse^13,14^.

Further studies have found an increase in depressive and generalized anxiety symptoms in pregnant women during the COVID-19 outbreak^15-17^. Reasons for this include existing mental illness, smoking, unplanned pregnancy, professional status, worries about loss of employment, domestic abuse, economic deprivation, and living in the UK and Ireland^10,12^.

COVID-19 secure measures implemented by UK hospitals in March 2020 to reduce viral transmission may have been extremely anxiety provoking for pregnant women. Restricting partner access to antenatal and ultrasound appointments, limiting antenatal and postnatal ward visits, and reducing the number of birth partners may have resulted in women feeling isolated and alone, thereby increasing anxiety.

The ‘Babies in Lockdown’ report^14^ surveyed 5474 parents; 34% of women who gave birth in lockdown stated that intrapartum care was not as planned, furthermore 68% reported that their ability to cope with pregnancy had been impacted by the COVID-19 pandemic^18^.

### Study objectives

This study aimed to investigate if anxiety in pregnancy increased during the COVID-19 pandemic and if moderate/high anxiety scores had an impact on maternal mental health, preterm birth and operative vaginal birth and/or cesarean section. Furthermore, the experiences of women who accessed maternity services during July 2020 to April 2021 were explored through semi-structured interviews to add context to quantitative data. Understanding the psychological implications of the COVID-19 pandemic on pregnant women will help to increase awareness and detection for women at risk of mental health deterioration and improve maternity care provision during future pandemics.

## METHODS

### Study design

This is a single-center, mixed-methods, observational cohort study to assess maternal anxiety during the COVID-19 pandemic and the impact on maternal mental health and birth outcome.

The primary outcome measure assessed antenatal and postnatal anxiety using self-reported scores from the Generalized Anxiety Disorder-7 (GAD-7) screening tool^19^. Secondary outcome measures assessed if women with moderate/high GAD-7 scores were more likely to develop a mental health condition, such as depression, anxiety, or any other mental illness during pregnancy or up to 6 weeks postnatally. The risk of preterm birth (<37 weeks gestation) and the need for operative vaginal birth or cesarean section, were also assessed by reviewing participant records at 6 weeks post-birth.

The GAD-7 questionnaire is a validated tool used to screen for Generalized Anxiety Disorder in the general population with a sensitivity of 0.83 (95% CI: 0.71–0.91) and specificity of 0.84 (95% CI: 0.70–0.92 at a cut-off point of 8)^20^. It has also been validated for use during pregnancy and is considered a clinically useful tool for the detection of generalized anxiety in the perinatal population (sensitivity of 61.3% and specificity of 72.7% at an optimal cut-off score of 13)^21^.

If used as a screening tool, further evaluation is recommended when the score is ≥10. Using the threshold score of 10, the GAD-7 has a sensitivity of 89% and a specificity of 82% for Generalized Anxiety Disorder. It is also moderately good for panic disorder (sensitivity 74%, specificity 81%), social anxiety disorder (sensitivity 72%, specificity 80%) and post-traumatic stress disorder (sensitivity 66%, specificity 81%)^22^.

A qualitative arm was included to explore the reasons for anxiety in women who scored moderately to highly on the GAD-7 during July 2020 to April 2021, which corresponds to the second and third waves of the pandemic in the UK^23^.

Mental health information, gestation at birth and mode of birth were reviewed and captured from the participant’s electronic patient records and by a follow-up telephone call at 6 weeks postnatally.

### Setting

This study was conducted at a North London NHS Trust across two hospital sites. Recruitment into the study took place between July 2020 and April 2021. Women were recruited into the study from inpatient antenatal wards or antenatal visits to the hospital in the second and third wave of the pandemic.

### Participants

A total of 206 eligible pregnant women (of any gestational age) were approached to participate in the study. A Patient Information Sheet (PIS) was provided to women explaining the study and time was allocated for questions. If the woman agreed to participate, signed informed consent was obtained by the researcher in accordance with Good Clinical Practice (GCP) guidance.

To eliminate the risk of selection bias, participants were randomly selected and recruited based on an inclusion criterion of no current mental illness (including anxiety) requiring medication, aged >18 years, not in established labor, and capacity to provide informed consent.

### Quantitative arm

To help understand anxiety scores in relation to COVID-19, participants who satisfied the inclusion criteria were asked two questions prior to completing the GAD-7. This was to assess if they had experienced anxiety prior to the COVID-19 pandemic and if they felt anxiety levels had increased since the COVID-19 pandemic. These questions were answered with a simple yes/no response. They were then asked to complete the GAD-7^19^. The GAD-7 score was calculated by assigning scores of 0, 1, 2, and 3, to the response categories of ‘not at all’, ‘several days’, ‘more than half the days’, and ‘nearly every day’, respectively for each question. The final score was then calculated by summing-up all scores for the seven questions. Scores of 5, 10, and 15, are taken as the cut-off points for mild, moderate and severe anxiety, respectively^19^. Table 1 provides details of GAD-7 response categories and scores.

**Table 1:** GAD-7 response categories and scores^19^, UK 2022 (N=194)

*Over the last 2 weeks, how often have you been bothered by the following problems?*	*Not at all*	*Several days*	*Over half the days*	*Nearly every day*
Feeling nervous, anxious or on edge	0	1	2	3
Not being able to stop or control worrying	0	1	2	3
Worrying too much about different things	0	1	2	3
Trouble relaxing	0	1	2	3
Being so restless that it’s hard to sit still	0	1	2	3
Becoming easily annoyed or irritable	0	1	2	3
Feeling afraid, as if something awful might happen	0	1	2	3

Scores are added for each column.

Women were informed of their overall score. If moderate (≥10) to high (≥15) scores were identified, a referral to the Women’s Health Counsellors was offered so that further support could be accessed if required. The woman’s midwife and GP were also informed, with consent.

All women were followed up at 6 weeks postnatally via a telephone call and asked to complete the GAD-7 questionnaire again for comparison between antenatal and postnatal scores.

### Qualitative arm

Women with moderate to high GAD-7 scores during the antenatal period were asked to participate in a semi-structured interview with a research midwife.

A separate PIS and consent form was used for women who agreed to participate in a semi-structured interview.

A social constructionist-interpretivist approach underpinned the design for the qualitative arm of this study. A social constructionist approach was the preferred theoretical framework to attempt to explain increased anxiety in pregnant women during the COVID-19 pandemic. Social constructionism assumes that reality is socially defined and builds on the subjective experience of everyday life. These qualitative data, therefore, attempt to understand the psychological impact of the COVID-19 pandemic in the pregnant population by drawing on the subjective experience of SARS-CoV-2 and the impact of social restrictions put in place to delay viral transmission, subsequently affecting maternity care.

Data were collected using a semi-structured interview guide. Key questions were used to explore why women felt anxious, how they were affected by the lockdown measures and their experiences of maternity care. Researchers followed the direction of discussion and probed where necessary to expand on the participant’s experiences to capture rich and meaningful data.

Six women participated in the interviews. Two were conducted face-to-face during antenatal appointments and 4 were conducted over the telephone and recorded using an encrypted National Health Service (NHS) mobile phone. Interviews were then transcribed verbatim by the researcher. All recordings were deleted after being saved securely to an encrypted memory device. Supplementary file provides details of semi-structured interview questions.

## RESULTS

### Statistical analysis

A total of 206 women were approached to participate in the study; 194 women completed the study (94%) while 12 (6%) declined to participate. Data were analyzed for all participants who completed the study (n=194). Participant baseline characteristics are provided in Table 2.

**Table 2:** Participant baseline characteristics, UK 2022 (N=194)

Characteristics	%
**Age** (years)	
≤35	81.5
>35	18
Mean age = 32 years	
**Mean gestational age at recruitment**	
30 weeks	
**Ethnicity**	
Black and minority ethnic groups (BAME)	38
Not BAME	48
Not stated	14
**Employment status and education level** (if stated)	
Employed	31
Unemployed	16
Professional qualification, higher degree or degree	34
A-level, BTECH	4.3
Unknown	6
**Medical or obstetric risk factors at the time of recruitment**	
No risk factors	68
BMI >35	5
BMI <18	0.4
Genital infections	1.4
Drug/alcohol issues	1.4
Gestational diabetes mellitus	1.4
Hypertension	1.4
Multiple pregnancy	1.4
Hypothyroidism	0.9
Migraines/headaches	0.4
Hepatitis B	0.4
Group B streptococcus infection	0.4
Non-English speaker	2.9

BMI: body mass index (kg/m^2^).

Quantitative data were analyzed using SPSS v27. Pearson’s correlation was used to examine the relationship between the COVID-19 pandemic and maternal anxiety and a repeated measures t-test was used to compare the mean antenatal (AN) and postnatal (PN) anxiety scores. Binary logistic regression was used to predict the risk of preterm birth and birth intervention with anxiety scores of ≥10.

Qualitative data were analyzed using NVIVO software for content thematic analysis. The data were coded for emerging themes to bring together similarities identified from women’s experiences.

### Quantitative results

**Figure 1:**
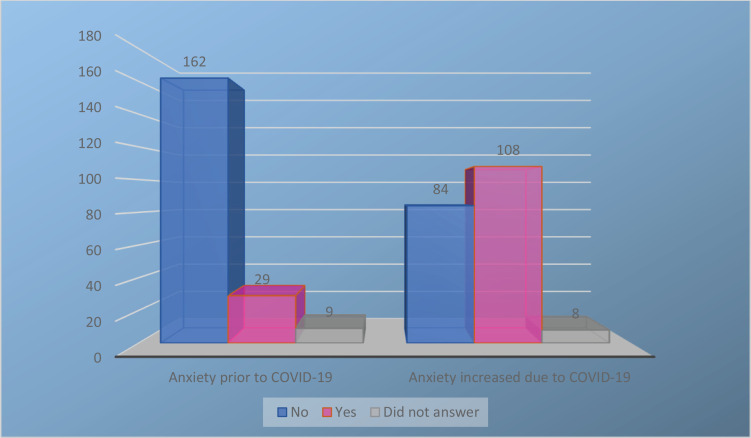
Maternal anxiety prior to and during COVID-19 pandemic result of two screening questions

Results from the two initial yes/no response questions, prior to completing the GAD-7, demonstrated that 67% of women felt an increase in anxiety during the COVID-19 pandemic. Only 29 women reported feeling anxious prior to the COVID-19 pandemic while 108 women reported increased anxiety because of the COVID-19 pandemic. A Pearson’s correlation analysis indicated there was a positive and significant correlation between COVID-19 and increased self-reported antenatal anxiety scores (r=0.47, n=194, p<0.001) (Figure 1).

A paired t-test^24^ was used to compare the mean GAD-7 scores antenatally (mean=5.30, SD=4.58) and postnatally (mean=3.77, SD=4.11).

The correlation between the two measures was estimated at r=0.445, p<0.001, suggesting that women who had higher anxiety scores in the antenatal period were more likely to score higher on the GAD-7 in the postnatal period. The t-test showed statistical significance in the difference between antenatal and postnatal mean GAD scores [t(193)=4.63; p=0.001; 95% CI: 0.87–2.16], with a higher mean score during the antenatal period. AN and PN GAD mean scores for COVID-19 waves two and three were very similar (Wave 3: mean=5.5 and Wave 2: mean=5.1, AN score) and (Wave 3: mean=3.5 and Wave 2: mean=3.4, PN score) suggesting that anxiety levels did not differ between waves 2 and 3 in this cohort of women.

Four (2%) of the women developed an anxiety disorder during the antenatal period with all four women scoring moderately to highly on the GAD-7 (Table 3).

**Table 3:** AN GAD-7 scores, gestation at birth and mode of birth, UK 2022 (N=194)

*AN GAD-7 scores*	*Number of participants*	*Gestation at birth*		*Mode of birth*	
	*n (%)*	*Duration (weeks)*	*n (%)*	*Mode*	*n (%)*
0–9 (mild anxiety)	158 (81.4)	>37	146 (75)	SVD	157 (80.9)
		<37	2 (1)	CS	1 (0.51)
10–14 (moderate anxiety)	27 (13.9)	>37	23 (11.8)	SVD	1 (0.51)
		<37	4 (2.06)	Instrumental	7 (3.60)
				CS	16 (8.24)
15–19 (severe anxiety)	10 (5.15)	>37	10 (5.15)	Instrumental	2 (1.03)
				CS	8 (4.12)

SVD: spontaneous vaginal delivery. CS: cesarean section.

Unadjusted logistic regression was used to examine if gestation of birth and mode of birth were associated with an anxiety score of >10 on the GAD-7. The model was statistically significant [χ²(4, n=184)=16.13; p<0.003] and able to differentiate between those with and without increased anxiety scores. The model explained between 8.4% (Cox and Snell R^2^) and 13.6% (Nagelkerke R^2^) of the variance in the dependent variable and correctly classified 81.5% of cases. However, neither birth gestation nor mode of birth significantly contributed to the model. Therefore, anxiety scores of >10 (moderate to high anxiety) do not appear to increase the likelihood of preterm birth or operative vaginal/cesarean section birth.

### Qualitative results

*Themes*

**Figure 2:**
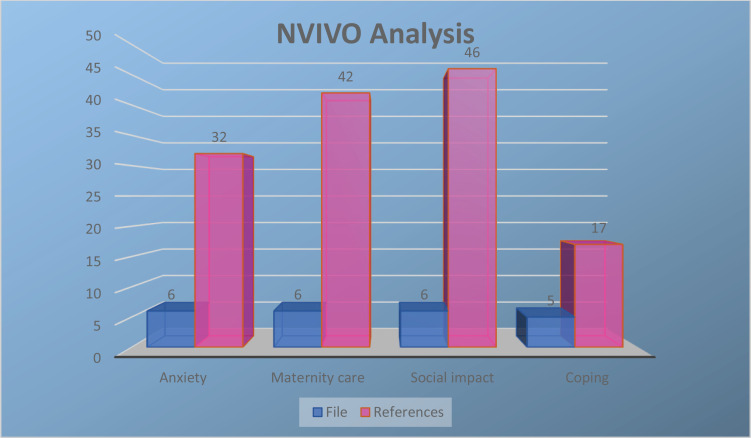
NVIVO analysis showing 4 themes and the number of files (participant interviews transcribed) and references (text taken from transcriptions and coded)

Four main themes were derived from data analysis, these were: anxiety, maternity care, social impact, and coping. Figure 2 shows the NVIVO analysis showing the 4 themes and the number of files (participant interviews transcribed) and references (text taken from transcriptions and coded). Four women were still pregnant at the time they were interviewed, and two women had already given birth. All had completed the GAD-7 antenatally and scored ≥10.

Participant characteristics for semi-structured interviews are given in the Supplementary file.

*Pregnancy anxiety*

The six women interviewed all admitted to feeling more anxious because of the COVID-19 pandemic. The following extracts provide details on specific factors that contributed to anxiety during pregnancy, taken from the first wave of the pandemic. Participants were concerned about contracting COVID-19 and how it may impact on their pregnancy:

*‘Well, it's quite a worrying time obviously because you want the baby to be ok, you just kind of think about the baby.’* (Participant 011)

*‘When I was pregnant, if we did come into contact with anything, like even when we went shopping and things like that, we were making sure that we were protecting ourselves as much as possible.’* (Participant 086)

One woman we interviewed was most anxious about labor and birth and what social distancing restrictions implemented by the hospital would mean for her and her partner:

*‘I was really worried about what it meant for the birth.’* (Participant 086)

*‘I was more concerned about whether it would mean my partner wouldn't be able to come to the birth.’* (Participant 086)

*‘… and also what would happen should me or my partner have COVID-19. Would that mean that I would then have to give birth in a different environment, in an environment that I hadn't planned for. Would I have to wear a mask when I was giving birth?’* (Participant 086)

Another participant discussed feeling anxious because her partner was not able to attend antenatal appointments:

*‘Cos like, I had to go to appointments all by myself, that was hard as well.’* (Participant 036)

*‘… and especially when it's like your first child you want your partner to be there throughout everything.’* (Participant 036)

*Maternity care*

Maternity care during the first wave of the pandemic followed frequently updated guidance by the Royal College Obstetricians and Gynecologists^25^. At the beginning of the pandemic this guidance was updated almost weekly depending on new evidence emerging.

Personal experience of maternity care captured through semi-structured interviews provided crucial insight to women’s experiences during the pandemic. What was most interesting were two opposing views on labor and birth and how two women discussed their very different experiences:

*‘It was really good and the staff were just really understanding generally, like they were good with my partner, and he wore a mask until someone said, oh you don't have to wear a mask, so it made him more comfortable too.’* (Participant 086)

*‘The staff all wore masks, bless them for the whole shift.’* (Participant 086)

The next participant describes her own experience which is less positive:

*‘I would say that there was a very big difference when I actually gave birth during COVID-19 [compared to first labor]. That was, for me, a really different experience. I think that my labor affected me just because of the way it was done different.’* (Participant 008)

*‘I wasn't used to having to support myself. I was like I need to support myself through this mentally and emotionally, and my partner couldn't be part of that process. I think my husband felt like he wasn't really there during it all.’* (Participant 008)

*‘It was very lonely I think, yeah.’* (Participant 008)

Participants also discussed their experiences of general maternity care:

*‘Everything was done very clinically, rather than human to human, but it's lockdown and there's like, limits to actual human [contact]. I dunno, maybe more phone calls or stuff like that-I think they did what they could at the time.’* (Participant 008)

*‘… and I actually wasn't told about all this stuff [changes to maternity care] that was gonna happen, erm, yeah that was a bit disappointing.’* (Participant 008)

*‘Yes, I was so worried, in fact, my first appointment they cancelled it, so I couldn't, you know, interact with my midwife.’* (Participant 024)

*Social impact*

The social impact on women was most apparent during analysis with this theme having the highest number of references. Women acknowledged a mixture of both positive and negative feelings towards the lockdown measures:

*‘Some days I felt bored, like there's nothing to do at home. Some days I felt happy cos I could like bake or do something to keep my mind occupied.’* (Participant 036)

One woman discussed how the COVID-19 pandemic impacted on her job and the anxiety this caused because of her pregnancy, while another woman described the relief at not having to commute:

*‘I work in retail and was in contact with a lot of customers, especially during Christmas and I felt like … it was dangerous for a pregnant woman to be in a retail environment, even with all the precautions.’* (Participant 085)

*‘I would say, like I said, just being at home has massively helped because I always used to find the tube quite a bit of a struggle.’* (Participant 086)

Another woman describes how she felt about explaining to her children that the world, as they knew it, was about to change:

*‘In the beginning it was a bit like everyone was at risk, as a mum I was like that was a bit scary in terms of how to deal with it not really for me but for the kids.’* (Participant 008)

One woman described how the lockdown had been positive for them and their family, and bought them closer together:

*‘We didn't spend much time together [as a family] so actually this lockdown and the lockdown starting in 2020 helped us to bond a lot.’* (Participant 085)

*Coping*

Women found various methods of coping with their anxiety during lockdown. Here women discuss how they coped:

*‘Well, my family helps a lot, I mean my husband and my son.’* (Participant 085)

*‘I think I ate more and I just like cooking.’* (Participant 036)

*‘Kind of try and get on with things and cope as best as I can. I don't really complain, I just keep going.’* (Participant 0011)

*‘I feel better already just talking to someone.’* (Participant 011)

## DISCUSSION

### Main findings

Interest into the psychological impact on maternal mental health in pregnancy has increased during the COVID-19 pandemic. The results from this study show a positive and significant correlation between the COVID-19 pandemic and increased antenatal self-reported anxiety scores. These findings were based on women’s answers to the two initial questions which asked about anxiety pre-COVID-19 and during COVID-19. Furthermore, there was a statistically significant difference between AN and PN mean GAD-7 scores suggesting that most women were more anxious during the antenatal period compared to the postnatal period.

### Interpretation

Although participants who scored more highly for antenatal anxiety sustained higher scores in the postnatal period, PN scores overall were lower. Women who scored higher on the GAD-7 antenatally did not appear to develop a mental health condition at 6 weeks postnatally. Women receiving pharmacological treatment for mental illness were excluded from this study; however, increased anxiety scores (≥10) were present in 18% (n=36) women in the antenatal period, and 9% (n=19) in the postnatal period. Consideration should be given to long-term follow-up, especially because risk of suicide in the first year after birth has been reported as the leading cause of maternal death in the UK^26^ and many cases are associated with untreated mental illness, such as depression which is commonly comorbid with anxiety^27^. This may be further exacerbated by the stress of giving birth during a pandemic alongside other social and financial challenges, such as fears about losing employment and increasing levels of domestic abuse^27^.

The results of this study support recent research showing similar findings on the emotional experiences of pregnant women during the COVID-19 pandemic in relation to increased anxiety^16,28,29^. Although this study did not demonstrate significant findings on how COVID-19 has impacted on mental health, being pregnant during a pandemic does increase the risk for poor mental health^16,28^. The most recent ‘Maternal Mental Health During a Pandemic’ report highlights the mental health challenges that women in pregnancy and early motherhood face^27^. Although 2% of women in this study developed tokophobia, anxiety and severe anxiety during pregnancy, these mental health issues did not appear to be related to the COVID-19 pandemic and three out of the four women had experienced miscarriage in the first trimester, which may give some insight towards their anxiety (Table 4).

**Table 4:** Characteristics of women who developed mental health issues during pregnancy, UK 2022 (N=194)

*Age (years)*	*Ethnicity*	*Parity*	*Miscarriage/ intrauterine death*	*Mental health issue*	*Details*
38	White (any other background)	G1 P0	No	Tokophobia	Extremely worried about mode of birth
38	Other (not stated)	G2 P0	Yes, first trimester	Anxiety	Related to previous miscarriage
26	Black (any other background)	G4 P2	Yes, first trimester	Severe anxiety	Developed after family bereavement
32	Other (any other ethnic origin)	G2 P0	Yes, first trimester	Tokophobia	Very anxious about vaginal birth

There was no increased risk of preterm birth or operative vaginal/cesarean section birth in women with higher GAD-7 scores (≥10). Previous studies have reported increased risk of preterm birth, low birth weight and birth intervention^2,4-9^ with maternal anxiety. However, it was noted that out of 194 women, those who scored >10 on the GAD-7 (n=36; 18%), 33 (92%) had mode of birth recorded as instrumental or CS, compared to only 0.63% (n=158) in the group scoring <10. Although, this was not statistically significant, high anxiety scores and impact on mode of birth may be worthy of further investigation.

### Strengths and limitations

A strength of this study is the inclusion of qualitative semi-structured interviews to help contextualize quantitative findings linked to GAD-7 anxiety scores ≥10. Semi-structured interviews are a widely used method of producing data in qualitative research^30^. In this study, semi-structured interviews were designed within a social-constructionist framework to investigate the influences of the pandemic on communal and individual life through the lens of pregnant women^31^. Through the qualitative analysis, it became apparent that women’s anxiety was fluid and on a parallel with day-to-day life experience. Four themes were identified: anxiety, maternity care, social impact, and coping; all in relation to the COVID-19 pandemic. Women discussed how societal changes during the pandemic had given rise to anxiety, especially in relation to maternity care and lockdown measures. It is undoubtedly clear that pregnant women require support during a pandemic, and that in part, they suffered from the indirect consequences of COVID-19, namely uncertainty in maternity care provision.

There are a number of limitations to this study. The first is that the overall sample size was small and secondly, women were only screened for anxiety at one time point during the pregnancy. Furthermore, we do not know if moderate to high anxiety scores remained or resolved for women as the pregnancy continued. Birth outcome was assessed using scores at the time of recruitment, regardless of gestational age. Therefore, to increase the reliability of results, the GAD-7 should be repeated at every antenatal contact to gain a true picture of overall anxiety scores during pregnancy to properly assess the impact on mental health and birth outcome in relation to persistent moderate to high anxiety levels in a larger sample size.

A case-control study with age-matched samples from non-pregnant populations was not undertaken; however, this may help to better understand if anxiety is increased more so in pregnancy than in the non-pregnant population.

Furthermore, COVID-19 vaccine-induced anxiety potentially adds another layer of complexity for pregnant women. In fact, one systematic review highlighted that globally, approximately 50% of pregnant women had increased anxiety about receiving a COVID-19 vaccine than contracting COVID-19 infection^32^, even though more than 275000 pregnant women have been vaccinated in the UK and the USA, with no concerning safety signals^33^. Therefore, further research is recommended to understand vaccine-induced anxiety during pregnancy.

## CONCLUSIONS

This study indicates an apparent increase in antenatal anxiety during the second and third wave of the COVID-19 pandemic. Qualitative data suggests that anxiety is predominantly related to maternity care and the social impact of lockdown measures. Further research is needed to establish if persistently high anxiety levels in the antenatal period adversely affect birth outcomes and contribute to the development of mental illness in the first year after childbirth. Healthcare professionals should be mindful that during a pandemic, pregnant women may be more vulnerable to increased anxiety; therefore, national antenatal care guidance should be updated to reflect the magnitude of this ongoing risk.

## Data Availability

The data supporting this research are available from the authors on reasonable request.

## References

[1] (2018). Mental health in pregnancy.

[2] Salehi L, Rahimzadeh M, Molaei E, Zaheri H, Esmaelzadeh-Saeieh S (2020). The relationship among fear and anxiety of COVID-19, pregnancy experience, and mental health disorder in pregnant women: A structural equation model. Brain Behav.

[3] Nakić Radoš S, Tadinac M, Herman R (2018). Anxiety During Pregnancy and Postpartum: Course, Predictors and Comorbidity with Postpartum Depression. Acta Clin Croat.

[4] Koelewijn JM, Sluijs AM, Vrijkotte TGM (2017). Possible relationship between general and pregnancy-related anxiety during the first half of pregnancy and the birth process: a prospective cohort study. BMJ Open.

[5] Laursen M, Johansen C, Hedegaard M (2009). Fear of childbirth and risk for birth complications in nulliparous women in the Danish National Birth Cohort. BJOG.

[6] Staneva A, Bogossian F, Pritchard M, Wittkowski A (2015). The effects of maternal depression, anxiety, and perceived stress during pregnancy on preterm birth: A systematic review. Women Birth.

[7] Khalesi ZB, Bokaie M (2018). The association between pregnancy-specific anxiety and preterm birth: a cohort study. Afr Health Sci.

[8] Grigoriadis S, Graves L, Peer M (2018). Maternal Anxiety During Pregnancy and the Association With Adverse Perinatal Outcomes: Systematic Review and Meta-Analysis. J Clin Psychiatry.

[9] Dowse E, Chan S, Ebert L (2020). Impact of Perinatal Depression and Anxiety on Birth Outcomes: A Retrospective Data Analysis. Matern Child Health J.

[10] Meijer JL, Bockting CL, Beijers C (2011). PRegnancy Outcomes after a Maternity Intervention for Stressful EmotionS (PROMISES): study protocol for a randomised controlled trial. Trials.

[11] Fitzgerald E, Parent C, Kee MZL, Meaney MJ (2021). Maternal Distress and Offspring Neurodevelopment: Challenges and Opportunities for Pre-clinical Research Models. Front Hum Neurosci.

[12] O'Donnell KJ, Glover V, Barker ED, O'Connor TG (2014). The persisting effect of maternal mood in pregnancy on childhood psychopathology. Dev Psychopathol.

[13] Holmes EA, O'Connor RC, Perry VH (2020). Multidisciplinary research priorities for the COVID-19 pandemic: a call for action for mental health science. Lancet Psychiatry.

[14] (2020). Royal College of Midwives and Royal College of Obstetricians and Gynaecologists joint policy statement on domestic abuse - November 2020.

[15] Papworth R, Harris A, Durcan G, Wilton J, Sinclair C (2021). Maternal mental health during a pandemic: A rapid evidence review of Covid-19's impact.

[16] Davenport MH, Meyer S, Meah VL, Strynadka MC, Khurana R (2020). Moms Are Not OK: COVID-19 and Maternal Mental Health. Front Glob Womens Health.

[17] Ceulemans M, Foulon V, Ngo E (2021). Mental health status of pregnant and breastfeeding women during the COVID-19 pandemic-A multinational cross-sectional study. Acta Obstet Gynecol Scand.

[18] (2020). Babies in Lockdown: listening to parents to build back better.

[19] Spitzer RL, Kroenke K, Williams JB, Löwe B (2006). A brief measure for assessing generalized anxiety disorder: the GAD-7. Arch Intern Med.

[20] Plummer F, Manea L, Trepel D, McMillan D (2016). Screening for anxiety disorders with the GAD-7 and GAD-2: a systematic review and diagnostic metaanalysis. Gen Hosp Psychiatry.

[21] Simpson W, Glazer M, Michalski N, Steiner M, Frey BN (2014). Comparative efficacy of the generalized anxiety disorder 7-item scale and the Edinburgh Postnatal Depression Scale as screening tools for generalized anxiety disorder in pregnancy and the postpartum period. Can J Psychiatry.

[22] Kroenke K, Spitzer RL, Williams JB, Monahan PO, Löwe B (2007). Anxiety disorders in primary care: prevalence, impairment, comorbidity, and detection. Ann Intern Med.

[23] (2022). Coronavirus (COVID-19) Infection Survey, UK: 20 May 2022.

[24] Posten HO, Rasch D, Tiku ML (1984). Robustness of the Two-Sample T-Test. Robustness of Statistical Methods and Nonparametric Statistics.

[25] (2021). Coronavirus (COVID-19), pregnancy and women’s health.

[26] (2022). New report on UK deaths during and after pregnancy.

[27] Khalifeh H, Hunt IM, Appleby L, Howard LM (2016). Suicide in perinatal and non-perinatal women in contact with psychiatric services: 15 year findings from a UK national inquiry. Lancet Psychiatry.

[28] Mappa I, Distefano FA, Rizzo G (2020). Effects of coronavirus 19 pandemic on maternal anxiety during pregnancy: a prospectic observational study. J Perinat Med.

[29] Pascal R, Crovetto F, Casas I (2022). Impact of the COVID-19 Pandemic on Maternal Well-Being during Pregnancy. J Clin Med.

[30] Green J, Thorogood N (2018). Qualitative Methods for Health Research.

[31] Galbin A (2014). AN INTRODUCTION TO SOCIAL CONSTRUCTIONISM. Social Research Reports.

[32] Carbone L, Di Girolamo R, Mappa I (2022). Worldwide beliefs among pregnant women on SARS-CoV-2 vaccine: a systematic review. Eur J Obstet Gynecol Reprod Biol.

[33] (2022). Coronavirus (COVID-19), pregnancy and women’s health.

