# Supplementary material for: Exploring social complexities of the COVID-19 pandemic on maternal anxiety: A mixed-methods observational cohort study
Source: Eur J Midwifery. 2022 Oct 11;6:63. doi: 10.18332/ejm/152200 (PMC9552077; doi:10.18332/ejm/152200)
Supplement: Supplementary file 1 [file EJM-6-63-s1.pdf]

Appendix

1. Semi-Structured Interviews investigating psychological wellbeing in pregnant women during the COVID-19 global pandemic

Interview time: 45 minutes

1) Do you think of yourself as an anxious person or do you usually feel anxious or often feel anxious?
2) In what way do you experience anxiety and have you ever had any psychological or medical help for anxiety?
3) Do you think being pregnant increased your anxiety? If so why do you think this is?
4) Do you think you started to feel more anxious because of COVID-19? If so what were you most worried about?
5) How do the government’s lockdown measures make you feel?
6) Is there anything you have done during the COVID-19 situation that has helped your anxiety?
7) Have you been offered any help during this time that has eased your anxiety?
8) Do you feel you were well supported by maternity services during the lockdown period?
If yes in what way were you well supported?
If not, how do you think you could have been better supported?
9) Do you feel you need further support to help you cope with the anxiety you are experiencing now during COVID-19?
10) Is there anything else you would like to say?

2. Participant characteristics for Semi-Structured Interviews (UK 2022, n=6)

Age	Ethnicity	Parity	Gestation at time of recruitment	GAD Score (AN)	GAD Score (PN)	Medical/Obstetric Complications	Mental Health History
35	White AOB	1	12+6	11	15	Hypothyroidism, Essential Hypertension	NIL
30	Not Stated	1	30+6	12	9	Low risk	NIL
36	Black AOB	1	26+1	16	5	Twins	NIL
30	South Asian	2	38+1	18	10	Hypothyroidism, Rheumatoid Arthritis	Anxiety & Depression
42	White AOB	1	25+5	19	6	Idiopathic heart palpitations	NIL
24	African Caribbean	0	37+3	15	12	Low risk	NIL

© 2022 Anderson M. et al.
